# Association of TYR SNP rs1042602 with Melanoma Risk and Prognosis

**DOI:** 10.3390/life12122004

**Published:** 2022-12-01

**Authors:** Arrate Sevilla, Ana Sánchez-Diez, Sofía Cobo, Neskuts Izagirre, Conrado Martinez-Cadenas, Rosa M. Martí, Teresa Puértolas, Blanca de Unamuno, José Bañuls, Rosa Izu, Jesús Gardeazabal, Aintzane Asumendi, María D. Boyano, Santos Alonso

**Affiliations:** 1 Department of Genetics, Physical Anthropology and Animal Physiology, Faculty of Science and Technology, University of the Basque Country/Euskal Herriko Unibertsitatea (UPV/EHU), 48940 Leioa, Spain; 2 Biocruces-Bizkaia Health Research Institute, 48903 Barakaldo, Spain; 3 Maria Goyri Building, Lab 2.08, UPV/EHU Scientific Park, Bizkaia Campus, 48940 Leioa, Spain; 4 Department of Dermatology, Basurto University Hospital, 48013 Bilbao, Spain; 5 Department of Medicine, Jaume I University of Castellon, 12071 Castellon, Spain; 6 Department of Dermatology, Hospital Universitari Arnau de Vilanova, 25198 Lleida, Spain; 7 Oncology Department, Hospital Universitario Miguel Servet, 50009 Zaragoza, Spain; 8 Department of Dermatology, Hospital Universitario de La Fe, 46026 Valencia, Spain; 9 Dermatology Department, Hospital General Universitario de Alicante, Instituto de Investigación Sanitaria y Biomédica de Alicante (ISABIAL), 03010 Alicante, Spain; 10 Dermatology Area, Clinical Medicine Department, Universidad Miguel Hernández, Sant Joan de Alicante, 03550 Alicante, Spain; 11 Department of Dermatology, Cruces University Hospital, 48903 Barakaldo, Spain; 12 Department of Cell Biology and Histology, Faculty of Medicine and Nursing, University of the Basque Country/Euskal Herriko Unibertsitatea (UPV/EHU), 48940 Leioa, Spain

**Keywords:** tyrosinase (*TYR*), melanoma, risk, prognosis, biomarker

## Abstract

Cutaneous melanoma is the most aggressive of skin tumors. In order to discover new biomarkers that could help us improve prognostic prediction in melanoma patients, we have searched for germline DNA variants associated with melanoma progression. Thus, after exome sequencing of a set of melanoma patients and healthy control individuals, we identified rs1042602, an SNP within *TYR*, as a good candidate. After genotyping rs1042602 in 1025 patients and 773 healthy donors, we found that the rs1042602-A allele was significantly associated with susceptibility to melanoma (CATT test: *p* = 0.0035). Interestingly, we also observed significant differences between patients with good and bad prognosis (5 years of follow-up) (n = 664) (CATT test for all samples *p* = 0.0384 and for men alone *p* = 0.0054). Disease-free-survival (DFS) analyses also showed that patients with the A allele had shorter DFS periods. In men, the association remained significant even in a multivariate Cox Proportional-hazards model, which was adjusted for age at diagnosis, Breslow thickness, ulceration and melanoma subtype (HR 0.4; 95% confidence interval (CI) 0.20–0.83; *p* = 0.0139). Based on our results, we propose that rs1042602-A is a risk allele for melanoma, which also seems to be responsible for a poorer prognosis of the disease, particularly in men.

## 1. Introduction

Cutaneous melanoma is the most aggressive of skin tumors due to its high metastatic capacity. Thus, it shows the highest mortality rate among skin cancer patients, although it only accounts for a small percentage of skin cancers. The incidence of melanoma is increasing worldwide, especially in fair-skinned individuals [1], and it is expected to increase in the next decades [2]. In this context, although early-stage melanomas show a tumor-specific 10-year survival of 75–95%, and about 90% of melanomas are diagnosed as primary tumors (without any evidence of metastasis at the time of diagnosis), patients with distant metastasis have a very poor prognosis [1]. Consequently, the early detection of melanoma is crucial for a good prognosis of the disease.

Based mainly on histopathological markers, the American Joint Committee of Cancer (AJCC) defines four main melanoma stages (stages I to IV) [3], which show different prognostic profiles. Breslow thickness and the presence of ulceration are considered the most important prognostic factors [1]. However, this staging strategy is not sufficient for a good prediction, as disease progression and survival can vary among individuals with same-stage primary tumors. Thus, the scientific community is actively engaged in discovering biomarkers that can improve the accuracy of predictions.

In this regard, a source of potential prognosis biomarkers can be found in germline DNA variants. Thus, Rendleman et al. (2013) [4] showed that germline variants previously associated with susceptibility to melanoma development were also correlated with disease progression. Since then, other authors have investigated this relationship [5], focusing both on melanoma susceptibility *loci* and other non-risk candidate genes, including genes involved in DNA repair, immune response, metabolism or cancer metastasis-associated pathways [6,7,8]. Thus, it seems likely that risk variants could also be used as recurrence or prognosis markers, and including those along with histopathological factors could improve disease outcome prediction.

It has long been known that some sequence variants in genes associated to skin pigmentation are also associated with a risk phenotype for melanoma [9,10,11,12]. However, in addition to risk, some germline variants in pigmentary genes have been also associated with melanoma clinicopathological features [13].

Thus, the aim of our study was to search for novel germline variants of pigmentary genes that while could have been described as risk variants for melanoma, could also be involved in melanoma progression, and consequently, could be used as prognostic biomarkers. For that purpose, by means of exome sequencing, we searched for genetic differences between melanoma patients and healthy controls. We then chose candidate variants in genes related to pigmentation and analyzed if those variants could be also associated with melanoma progression.

## 2. Materials and Methods

### 2.1. Ethics Statement

The study protocol conformed to the tenets of the Declaration of Helsinki (Version Brazil 2013) and was approved by the Euskadi Ethics Committee (CES-BIOEF 2019-16) and by the corresponding Committee of each of the Biobanks that contributed samples. All patients and healthy donors gave written informed consent to participate in this study.

### 2.2. Patients and Samples

In this study, a total of 1025 patients were included (540 women and 485 men; see Table 1), all of them with histologically confirmed malignant melanoma. Patients were untreated, other than primary surgery. Disease stages were classified according to the 8th edition of AJCC (American Joint Committee on Cancer) [3]. In addition, a total of 773 healthy individuals were also included as controls. For some analyses, only patients with at least 5 years of follow-up were considered (n = 664, 360 women and 304 men; see Table 2). Patients were divided into two groups according to disease prognosis: patients with good prognosis (without metastasis in the following 5 years after diagnosis of the primary tumor) and metastatic patients (patients who developed metastasis after surgery and within 5 years after diagnosis).

Several biobanks integrated into the Spanish National Biobanks Network contributed samples and data to this study, which were processed following standard operating procedures with the appropriate approval of the Ethics and Scientific Committees.

These biobanks were the following: the Basque Biobank (B.0000140), the ISABIAL Biobank (B.0000834) (also integrated into the Valencian Biobanking Network), the IRBLleida Biobank (B.0000682, PT20/00021) (also integrated into Xarxa de Bancs de Tumors de Catalunya (XBTC)), the Navarrabiomed Biobank (B.0000735, PT17/0015/0007), the La Fe Biobank (B.0000723, PT20/00179), the Castellon Province Hospital Biobank (BI/14/2018) and the Aragon Health Sciences Institute, in the framework of the Biobank of Aragon (B.0000873, PT20/00112).

The clinical and diagnostic data for each patient were collected retrospectively or provided by some of the Biobanks. For patients with multiple melanomas, the melanoma with the highest AJCC stage was included.

DNA from healthy donors was isolated from saliva, and DNA extraction was performed following a standard phenol–chloroform protocol (approved by the bioethics committee of UPV/EHU, number M10_2015_243).

### 2.3. Exome Analysis 

Exome sequencing was performed on DNA isolated from peripheral blood of patients with melanoma (n = 27) by means of the Illumina platform (MiSeq) in the Genomics Services Facilities (SGIKER) of UPV/EHU. Libraries were obtained with the TrueSeq Exome kit (Illumina). We obtained 2 runs per sample, with 75 bp pair-ended reads. The average depth per sample was 26x and the average number of reads per sample was 31.3 M.

Alignment of reads was completed against the reference genome hg19 by means of BWA v0.7.12-r1039. After the alignment, the two runs per sample were merged by means of GATK MergeSamFiles (v.4.2.6.0). Duplicated reads were marked with GATK MarkDuplicatesWithMateCigar (v.4.2.6.0). Systematic errors in the sequencing process were corrected by running GATK Base Quality Score Recalibration (BQSR) (v.4.2.6.0).

Variant calling was performed on the recalibrated files following the GATK best practices. Firstly, HaplotypeCaller was used with individual samples, in gVCF mode. Then, all samples were aggregated in a datastore by means of GenomicsDBImport. Next, a joint genotyping of all samples with GenotypeGVCFs was performed. To filter out possible false positives, a Variant Quality Score Recalibration was applied to the data. All variants in which the GQ value was less than 20 and/or a DP value less than 8 in any individual were removed. 

Exomes from melanoma patients were compared with the exomes of healthy individuals from the Basque Country (n = 26) (previously analyzed by our group; manuscript in preparation), which were used as healthy controls. 

#### Comparison of Allele and Genotype Frequencies between Melanoma and Control Samples

To check for allele frequency differences between healthy controls and melanoma patients, we used Weir and Cockerham’s (1984) estimator [14] as implemented in VCFtools [15]. Furthermore, we also used the Cochran–Armitage test for trend to compare genotype frequencies by means of R package ‘MaXact’ 0.2.1 [16].

In addition, a PCA was performed with our melanoma and control samples, along with the Eurasian samples from the 1000 Genomes Project (1000 GP): FIN: Finnish in Finland; CHS: Han Chinese South; CHB: Han Chinese in Beijing, China; JPT: Japanese in Tokyo, Japan; CEU: Utah residents (CEPH) with Northern and Western European ancestry; IBS: Iberian populations in Spain; TSI: Toscani in Italy. For that, we selected the SNPs overlapping our exomes SNPs. PCA was performed by means of the R package SNPRelate v1.28.0 [17].

### 2.4. SNPs Genotyping

A SNP from *TYR* (rs1042602) was initially genotyped in a set of 100 melanoma patients and 100 healthy individuals. Genotyping was performed by means of RT-qPCR in a StepOne Real-Time PCR System (Applied Biosystems, Waltham, MA, USA), using TaqMan SNP Genotyping Assays, TaqPath™ ProAmp™ Master Mix (Applied Biosystems, Waltham, MA, USA) and 30 ng of genomic DNA. Thermocycling conditions were as follows: a first step at 95 °C for 5 min to activate the enzyme, followed by 40 cycles of 95 °C for 15 s and 60 °C for 1 min, and a final step of 60 °C for 30 s.

As rs1042602 yielded significant differences between the two extended groups, it was finally analyzed in a total of 1025 patients and 773 controls to confirm the differences.

### 2.5. Statistical Analysis of Association

Differences in the genotypic frequencies between patients and controls, and between patients with good and bad prognosis, were analyzed by means of Fisher Exact Test (or Chi-Square Test) and Cochran–Armitage test for trend (CATT) (one-sided, dominant model (theta = 1), using the R package ‘MaXact’ 0.2.1 [16].

### 2.6. Disease-Free Survival (DFS) Analysis 

We defined the disease-free interval as the period between the date of surgical excision of the primary tumor and the date of the appearance of metastasis or the last date of follow-up.

Survival curves based on Kaplan–Meier methods and the Cox Proportional-hazards model were used to calculate disease-free survival outcomes and to investigate differences with respect to the rs1042602 genotype (CC, CA or AA). Kaplan–Meier curves were drawn using the ‘survfit’ routine in the R package ‘survival’ [18]. The statistical significance of differences between survival curves was determined by using the non-parametric log-rank test (*p*  <  0.05). We also analyzed differences in survival distributions by the Cox Proportional-hazards model. Models were fitted using the ‘coxph’ routine in the R package ‘survival’. To assess the influence of the rs1042602 genotype, we performed univariate Cox logistic regression analysis and multivariate analysis adjusted for sex, age of diagnosis, Breslow thickness, ulceration and histological subtype (if covariates were significant in univariate analyses). The hazard ratio (HR) for each of the survival outcomes was also calculated. Beta value was also obtained using the glmm.wald test in the GMMAT R package v1.3.2 [19].

## 3. Results

A total of 65,221 SNPs were observed in our set of 27 exomes from melanoma patients. The Ti/Tv ratio was 2.87, which indicates good quality. Based on the results of Weir and Cockerham’s FST, we observed a total of 644 different genes for which different SNPs showed high Weir and Cockerham’s FST values (above the 95th percentile) between the melanoma sample and the control sample. This list of genes showed enrichment in Jensen disease terms such as melanoma (p-adj 5.73 × 10^−16^) or skin cancer (p-adj 7.93 × 10^−18^). In order to add biological meaning to the results, we focused on those genes involved in pigmentation. We detected Weir and Cockerham’s FST-significant SNPs in genes such as *TYR* (rs1042602), *DCT* (rs755684), *HERC2* (rs61756153) or *SLC45A2* (rs16891982). Results of the Cochran–Armitage test for trend (CATT) indicated that for theta = 1, the enrichment in Jensen diseases was highest for melanoma and skin cancer (skin cancer at the top position, along with liver cancer, adjusted *p*-value = 1.10 × 10^−9^, and melanoma on second position, along with breast cancer, adjusted *p*-value = 1.30 × 10^−9^). Two pigmentation genes showed (nominal) significant SNPs under a CATT model with theta = 1, *TYR* (rs1042602; *p*-value = 0.027) and *HERC2* (rs61756153; *p*-value = 0.039).

We decided to focus on rs1042602, because this SNP shows the smallest *p*-value in CATT and because tyrosinase (*TYR*) is the rate-limiting enzyme in melanogenesis. This SNP (rs1042602) is a C/A missense variant at chr11:88911696 (hg19), and it has been previously associated with different pigmentary phenotypes, such as skin pigmentation [20,21,22,23,24,25], eye color [26], hair color [23,27] and absence of freckles [28,29].

Consequently, to confirm that the signal from this initial exploration was not spurious, we subsequently genotyped rs1042602 in a new set of 100 patients and 100 healthy controls. Once we confirmed that differences in allele’s frequencies were significant (by Fisher Exact Test and CATT test, *p*-value < 0.05), we genotyped rs1042602 in an additional set of individuals, making up a total of 1025 melanoma patients and 773 healthy controls genotyped. 

**Figure 1:**
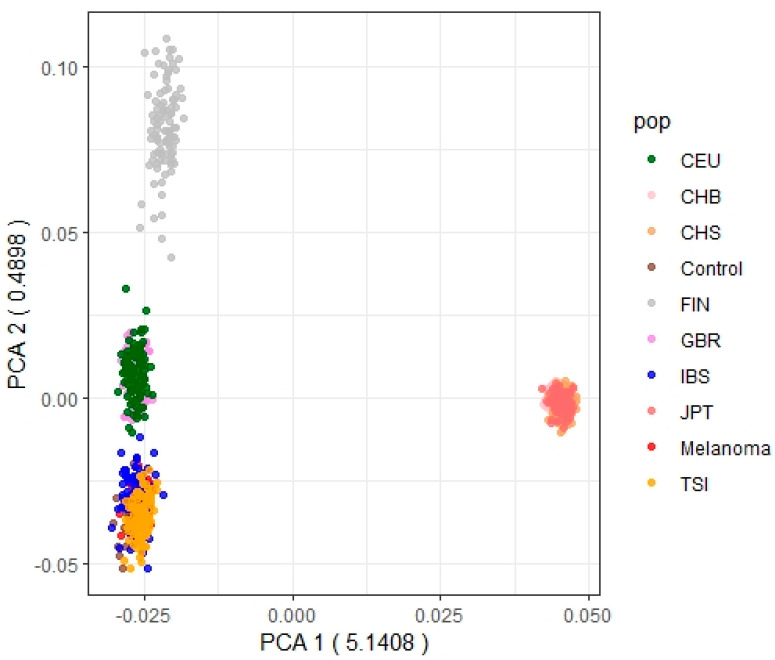
Principal component analysis of the control samples (control), the melanoma samples (melanoma), and the Eurasian samples from the 1000 G project (FIN: Finnish in Finland; CHS: Han Chinese South; CHB: Han Chinese in Beijing, China; JPT: Japanese in Tokyo, Japan; CEU: Utah residents (CEPH) with Northern and Western European ancestry; IBS: Iberian populations in Spain; TSI: Toscani in Italy).

Patients had a median follow-up of 63 months (298 patients with metastasis and 727 with good prognosis). Clinicopathological characteristics of patients are summarized in Table 1. As our patients’ samples were collected from different hospitals across Spain, in order to check if possible sample heterogeneity could be influencing our results, we conducted a Principal Component Analysis (PCA) with our melanoma samples, our control samples and the Eurasian samples from the 1000 Genomes Project (1000 GP). Figure 1 shows that our melanoma samples cluster with the general Iberian population of the 1000 GP and the rest of the Basques control samples. Therefore, differences do not seem to be large enough to have an effect on our results. After confirming this, we again observed that there was a statistically significant difference between the frequencies of the genotypes between patients and controls (Chi-Square Test: *p*-value = 0.0044; CATT test: *p*-value = 0.0035), with melanoma patients showing higher frequencies for the AA genotype (Table 3). However, when stratified by sex, the difference was only significant for men (Chi-Square Test: *p*-value = 0.0015; CATT test: *p*-value = 0.0030) but not for women (Chi-Square Test: *p*-value = 0.5915; CATT test: *p*-value = 0.2061) (Table 3).

Then, we decided to split the patients into two groups according to disease prognosis: “non-metastatic patients” (patients with good prognosis) and “metastatic patients” (patients who developed metastasis within 5 years after surgery for primary tumors), and we compared genotype frequencies for rs1042602 between those two groups. For this analysis, we only included patients with a follow-up of at least 5 years (n = 664): 195 metastatic and 469 non-metastatic patients. Interestingly, we also observed significant differences between the two groups (men and women together) with the CATT test (Chi-Square Test: *p*-value = 0.1526; CATT test: *p*-value = 0.0384) and only for men with both tests (Chi-Square Test: *p*-value = 0.0308; CATT test: *p*-value = 0.0054), but not for the ‘only-women’ group (Chi-Square Test: *p*-value = 0.7118; CATT test: *p*-value = 0.5968) (Table 4). The differences were also statistically significant when only melanoma stages I, II and III were included (both in men and when all samples are considered). The differences were not significant with stage II samples only, but the trend was similar (higher frequency of AA genotype and lower frequency of CC genotype), particularly in men (Table 4 and Table 5). 

In addition, a generalized linear mixed model (GLMM)-based Wald test also showed statistically significant differences in men (*p*-value = 0.012). This test inferred a beta-value of 0.89. In this case, we considered AA and AC genotypes versus CC genotype. Thus, having the A allele in homozygosis or heterozygosis would have an effect on the outcome. 

**Figure 2:**
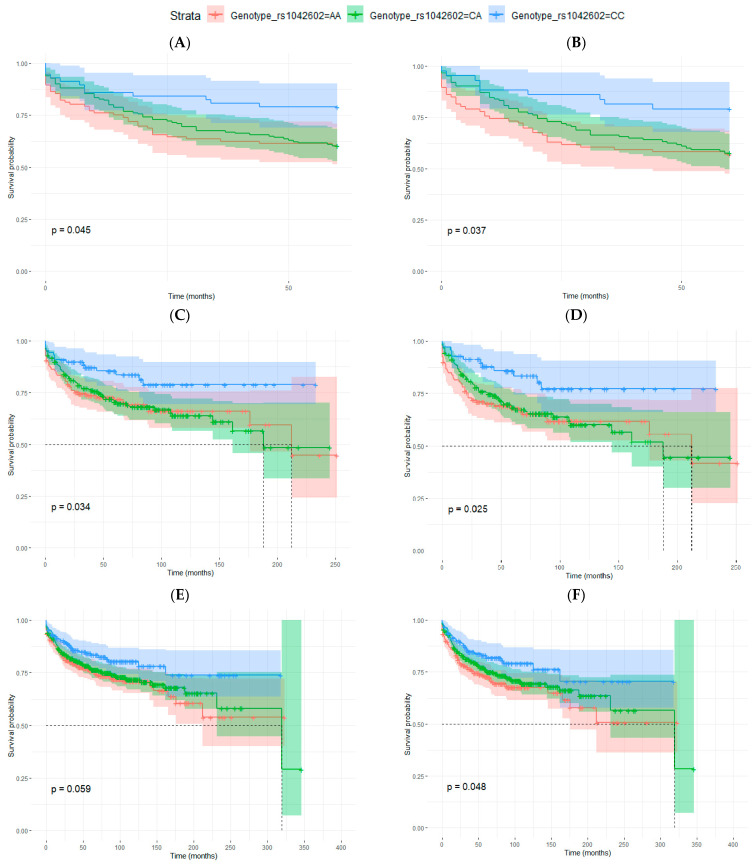
Kaplan–Meier curves for disease-free survival (DFS) analysis in relation to rs1042602 genotype. (**A**,**B**) men with 5 years of follow-up: (**A**) all AJCC melanoma stages (*p*-value = 0.045) and (**B**) considering only stages I, II and III (*p*-value = 0.037), (**C**–**F**) patients with any follow-up period: (**C**) men, all AJCC melanoma stages (*p*-value = 0.034) (**D**), men, stages I, II and III (*p*-value = 0.025), (**E**) men and women, all AJCC melanoma stages (*p*-value = 0.059) and (**F**) men and women, stages I, II and III (*p*-value = 0.048). Log-rank test *p*-values are also indicated in each figure.

To further analyze the possible relation of the A allele of rs1042602 and the development of metastasis, we evaluated the impact of the genotype for rs1042602 on disease-free survival (DFS). Considering patients with 5 years of follow-up, we observed statistically significant differences in men for the CC genotype (considering male patients with all AJCC melanoma stages and when only stages I, II and III were considered) (Table 6, Figure 2). rs1042602 was associated with DFS even after multivariate analyses, and it was adjusted for age at diagnosis, Breslow thickness, ulceration and melanoma subtype (for all stages: HR 0.41; 95% confidence interval (CI) 0.20–0.83; *p*-value = 0.0139 and for I-III stages: HR 0.36; 95% CI 0.16–0.79; *p*-value = 0.0107) (Table 6).

When all samples were considered in the analyses (median follow-up of 63 months), differences were significant for men (also in multivariate analyses) and also for all samples together (men and women) for melanoma stages I, II and III (Table 6, Figure 2) but not for women. In general, in all analyses, patients with CC genotype had longer disease-free survival periods, but the survival curves of patients with AA and CA genotypes were similar. 

**Figure 3:**
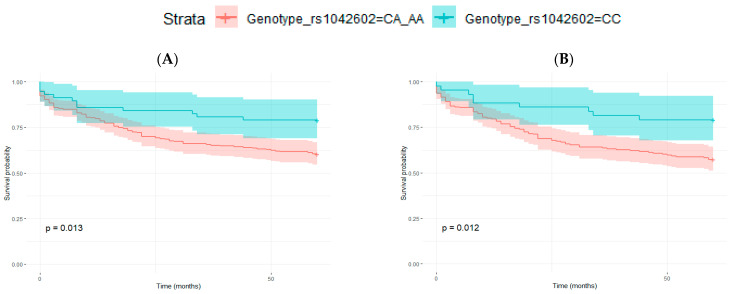

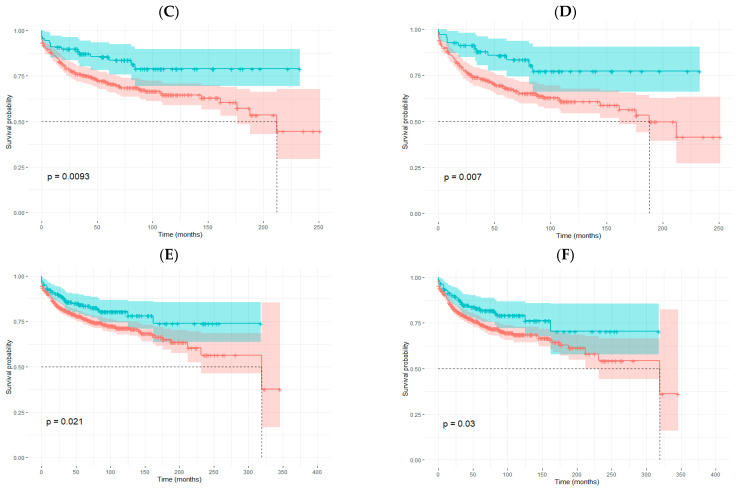
Kaplan–Meier curves for disease-free survival (DFS) analysis in relation to rs1042602 genotype, grouped as CC vs. CA and AA. (**A**,**B**) men with 5 years of follow-up: (**A**) all AJCC melanoma stages (*p*-value = 0.013), and (**B**) considering only stages I, II and III (*p*-value = 0.012), (**C**–**F**) patients with any follow-up period: (**C**) men, all AJCC melanoma stages (*p*-value = 0.009), (**D**), men, stages I, II and III (*p*-value = 0.007), (**E**) men and women, all AJCC melanoma stages (*p*-value = 0.021) and (**F**) men and women, stages I, II and III (*p*-value = 0.030). Log-rank test *p*-values are also indicated in each figure.

Differences in the disease-free survival periods were greater when patients were grouped as those with CC genotype versus those carrying A allele (CA and AA). This is in line with the election of the dominant model (theta = 1) in the Cochran–Armitage test for trend (CATT), which assumes a dominant relationship between the alleles. Thus, having one A allele is enough to have an effect on the outcome. In this case, differences between Kaplan–Meier curves were statistically significant (log-rank tests) for all samples together and for men in all the analysis performed (considering patients with 5 years of follow-up or all patients with I, II and III melanoma stages or all melanoma stages) (Figure 3). However, in the multivariate Cox Proportional-hazards model, CC genotype remained significantly associated to DFS only in men (Table 7).

**Figure 4:**
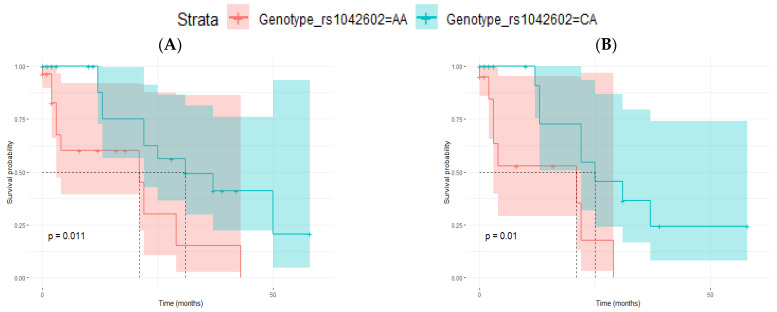
Kaplan–Meier curves for distant metastasis-free survival (DMFS) analysis in relation to rs1042602 genotype (CA vs. AA) in patients with stage III melanomas and 5 years of follow-up. (**A**) Men and women (*p*-value = 0.011) and (**B**) men (*p*-value = 0.010). Log-rank test *p*-values are also indicated in each figure.

Among patients with stage III melanomas, the frequency of the CC genotype was low (11.7%). In contrast, the frequency of the AA genotype was higher (40.3%) than in patients with stage I and II melanomas. This difference in the frequencies was greater in men: frequency of CC: 6.1% and AA: 44.9% (Table 8). As the number of patients with stage III melanomas and CC genotype was low, we decided in this case to analyze the outcome of patients with either CA or AA genotypes only. All patients with stage III melanomas have regional lymph node metastases at diagnosis (it is a key parameter for the AJCC staging). To analyze if the rs1042602 genotype could have an influence on the development of distant metastasis, we performed a ‘distant metastasis-free survival’ (DMFS) analysis comparing patients with stage III melanomas and a follow-up of 5 years with or without distant metastasis (only patients with CA and AA genotypes). Although the sample size was small, we observed that patients with AA genotype had shorter DMFS periods in all samples (n = 71) and in men (n = 45) (Table 9, Figure 4). 

Patients with stage II melanomas also showed a tendency toward lower CC frequencies and higher AA frequencies (Table 8), but there were not differences in the length of DMFS periods.

## 4. Discussion

Early diagnosis of melanoma is crucial for the survival of the patients. Fortunately, most melanomas are detected in early stages, which results in a better prognosis. However, even if the melanoma tumor is detected at early stages, many patients still develop metastasis during the follow-up period, even after the surgical removal of the primary tumor. Breslow thickness and histological ulceration are the main parameters for the diagnosis and prognosis [3]. However, they are not always sufficient for an accurate diagnosis and especially for a reliable prognosis, and consequently, additional biomarkers are needed. Interestingly, association analyses have identified germline variations associated not only with susceptibility or protection against melanoma but also with disease prognosis [4,5]. 

In this context, common polymorphisms in pigmentary genes should be considered [11], as these genes contain many risk polymorphisms that might also influence melanoma prognosis. In fact, less pigmented or amelanotic melanomas tend to have a more aggressive phenotype [30,31,32]. 

Thus, we searched for candidate SNPs by comparing the exomes of melanoma patients and healthy controls. We found that one SNP, rs1042602, in the pigmentary locus *TYR*, could be associated with melanoma susceptibility and prognosis.

rs1042602 (c.575C>A), first found in patients with Oculocutaneous Albinism [33], is a missense variant within the *TYR* locus, which codes for tyrosinase, a key enzyme that controls the first steps in melanogenesis [34]. This non-synonymous substitution leads to an amino acid change (S192Y) in the catalytic site of tyrosinase and has been related to a lower enzymatic activity, lower tyrosinase content [35,36], a reduced expression of *TYR* and lower melanin content [24].

The alternative A allele shows the highest frequency in Europeans (0.37), and it is nearly absent in East Asia (0.001) and Africa (0.012). It is present at intermediate frequencies in South Asian populations (0.063) and also in admixed populations from South America (0.238) (the 1000 Genomes Project, phase 3 data). In our control samples from Spain, the frequency of the A allele was 0.5. In Spanish melanoma patients, the frequency of the alternative A allele was slightly higher (0.54) than in controls.

It has been reported that the A allele of rs1042602 has been associated with lower skin pigmentation levels in different populations [20,21,22,23,24,25,37], and it is broadly accepted that this allele is also associated with lighter skin pigmentation. In fact, it has been used to predict skin color [38]. It has also been associated with other pigmentary traits, such as hair color [23,27], eye color [26] or the absence of freckles [28,29].

The 192Y variant is also associated with albinism [39], especially in combination with the R402 variant in the same locus. Due to this association, some authors argue that it should actually be considered a pathogenic variant [40,41].

Regarding melanoma susceptibility, rs1042602-A has been proposed as a risk variant for melanoma [42,43,44]. Bishop et al. (2009) [42] first reported rs1042602 to be related to melanoma risk in a genome-wide association study in populations of European ancestry living at different latitudes. However, other authors failed to find an association with melanoma susceptibility [45,46]. These discrepancies in the results could be due to the different geographical origin of the sample sets. The study of Duffy et al. (2010) [45] was performed with samples exclusively from Australia. Although they reported having diverse European ancestries, the sample was mostly of North European origin. Castaneda-García et al. (2022) [46] only included British samples in their study (1959 melanoma cases and 737 controls). According to data from the 1000 Genomes Project, the frequency of the non-reference A allele in British and other North European populations is lower than in South European populations such as the Spanish and the Italian. In contrast, Bishop et al. (2009) [42] used a wider sample set, including patients from Australia, France, Italy, Spain, Netherlands, Sweden and the UK (1650 melanoma cases and 4336 controls), and they performed replication in 2 additional cohorts.

In our samples from Spain, we genotyped rs1042602 in 1025 patients and 773 controls, and we do observe statistically significant differences between patients and healthy controls. Thus, our data are in line with the results by Bishop et al. (2009) [42] and Hernando et al. (2016) [44], and we confirm that rs1042602-A is a risk allele for melanoma that should be taken into account. 

However, when stratified by sex, the association was only significant for men, suggesting that the rs1042602 influence on melanoma susceptibility could be dependent on sex. In fact, this SNP has been reported to have different allelic effects by sex [44]. Thus, Hernando et al. (2016) [44] observed that rs1042602 was associated with nevi count and eye color and also with melanoma risk only in males, which is in agreement with our results. Several authors had proposed that germline melanoma risk variants could also be involved in the evolution of the disease [4,8]. Then, once we confirmed that rs1042602 is a risk SNP, we decided to analyze if it could also be associated with prognosis. For that purpose, we selected patients with a follow-up of 5 years after surgery of primary tumor and made two groups according to their prognosis. 

Interestingly, we observed a statistically significant difference between genotypic frequencies for rs1042602 between metastatic and non-metastatic patients. The difference was significant only for men but not for women. In addition to that, we further focused on patients with stage II melanomas, because these patients show the greatest variability with respect to evolution of the disease. In this sense, only a small percentage of patients with ‘in situ’ and stage I melanomas develop metastasis, and patients with stage III and IV melanomas have already lymph node or distant metastasis, respectively, at the moment of the diagnosis, and are under supervision. Consequently, patients with stage II melanoma would benefit most from a more accurate prediction, as clinical decisions could be made well in advance. However, our results only considering stage II melanomas did not reach statistical significance, which was probably due to the small sample size. It would be worth analyzing this SNP in a wider sample of patients with stage II melanomas. 

We also found that in our Spanish sample, rs1042602 was associated with disease-free survival (DFS). Homozygous patients for the reference allele (CC) had a longer disease-free survival period compared with heterozygotes (CA) and homozygotes for the alternative allele (AA). We initially performed the survival analyses with patients with 5 years of follow-up in order to minimize variability and bias regarding monitoring. However, once we observed the significant differences between DFS in those patients, we repeated the analysis with all the patients (median follow-up of 63 months), and differences were still significant both for men and for all samples together (men and women) considering melanoma stages I, II and III. These results show that carrying one risk allele of rs1042602 is enough to have an influence on the disease-free survival time period of melanoma patients. This correlates with the proposed functional consequence of the A allele, which is reported to decrease tyrosinase activity by 40% [35,36].

As survival curves for patients with CA and AA genotypes were similar, we performed survival analyses grouping patients carrying the A allele versus homozygotes for the C reference allele. We confirmed again that patients carrying one or two copies of the A allele had shorter disease-free survival periods. Actually, rs1042602 remained associated with DFS in men even after adjusting for the main predictive parameters in melanoma, including Breslow thickness, ulceration, melanoma subtype and age at diagnosis.

An interesting observation is that in patients with stage III melanomas, the proportion of individuals with the CC genotype was very low, and the frequency of AA was higher than in other patients. All patients with stage III melanomas have node metastases at the moment of the diagnosis. In this context, we compared the outcome of patients with CA versus AA genotypes, as regards the development of distant metastasis. Patients with the AA genotype showed shorter disease-free periods considering distant metastasis. Although the sample size was small, our data suggest that the A allele could also be a risk allele for the development of distant metastasis in stage III melanoma patients.

The possible explanation behind the association of rs1042602 and melanoma risk could be explained if we consider that a less active tyrosinase could lead to a light-skin phenotype and consequently to a “melanoma risk phenotype”.

On the other hand, we know that in general, pigmentation loss in melanomas is associated to poorer prognosis; thus, hypopigmented and amelanotic melanomas tend to be more aggressive and patients have shorter survival rates [30,31,32]. However, the fact that amelanotic melanomas tend to be diagnosed at more advanced stages also influences disease outcome [30].

Independently of tyrosinase’s pigmentary function, the loss or reduction in tyrosinase activity could also benefit tumor growth and metastasis. Tyrosinase is known to be a melanoma-associated antigen [47,48]; consequently, its loss would be beneficial for tumor escape from the immune system. Other authors reported that tyrosinase downregulates cell migration, and recent works suggest that tyrosinase may have anti-tumoral properties. Fürst et al. (2019) [49] also observed that hypopigmented melanomas were more aggressive and that it was related to tyrosinase degradation induced by DNp73. This loss of tyrosinase led to the reactivation of the EMT (epithelial–mesenchymal transition) signaling cascade; as a result, cells acquire a mesenchymal-like cell phenotype and, consequently, become more invasive. Thus, a new function was proposed for tyrosinase as an EMT inhibitor. Furthermore, Kamo et al. (2022) [50] demonstrated that tyrosinase is able to suppress vasculogenic mimicry (VM) in human melanoma cells, avoiding the formation of a vascular-like network that provides tumors with oxygen and nutrients. Both EMT and VM are directly related to the metastatic potential of tumors. Thus, if tyrosinase activity is lost or decreased, melanoma would more easily grow and invade other tissues. 

Our results also indicate that the association of rs1042602-A with a worse prognosis may be dependent on the sex of the patient, since when sex is considered in the analyses; association is only significant for men. This difference between males and females could be explained by the characteristics of the patients and/or the melanomas. In general, men tend to be diagnosed at more advanced ages (median 62 years versus 56 years in women in our sample). The location of the primary tumor is also relevant. In our samples, among men, more than 50% of melanomas are located in the trunk, while in women, the main location is lower limbs (32%) and secondly trunk (31%). In men, the percentage of head and neck melanomas is also higher than in women. Prognosis is known to be worse in male patients with increased age and in trunk and head-and-neck tumors compared to melanomas on the limbs [1].

It would be interesting to analyze if UV irradiation is related to the effect of rs1042602 on melanoma progression. Melanomas are classified into two groups with respect to their development pathway as either nevogenic or associated with cumulative solar damage [51]. Unfortunately, we do not have data about the amount of solar damage in our patients or about high-depth sequencing data that could show a UV radiation mutation signature. As different body locations have variable levels of sun exposure, we tried to analyze it indirectly by analyzing the association of rs1042602 and melanoma progression separately in head and neck melanomas (as an approach for sun-exposed regions) and in truncal melanomas (as unexposed regions). The association was significant for head and neck melanomas (n = 158) but not for trunk melanomas (n = 424). This preliminary approach suggests that it is worth analyzing if sun exposure is also modulating the effect of rs1042602 on melanoma outcome.

## 5. Conclusions

Based on our results, we propose that rs1042602 is not only a risk allele for melanoma, as it has previously been claimed; but, in addition to that, the A allele would also be associated with a poorer prognosis of the disease, particularly in men. 

Together with histological parameters and additional biomarkers, rs1042602 genotyping could help obtain a more accurate prediction of the evolution of the disease. Obviously, our results need to be replicated in additional cohorts. If it is confirmed that rs1042602-A is a bad prognostic factor, it could then be constituted as a powerful biomarker that could be easily implemented in a clinical context with a minimally invasive protocol and from small amounts of DNA.

## Figures and Tables

**Table 1:** Clinicopathological features of melanoma patients (n = 1025). SSM, superficial spreading melanoma; NM, nodular melanoma; LM, lentigo maligna; LMM, lentigo maligna melanoma; ALM, acral lentiginous melanoma; nd: no data.

	All Patients	Women	Men
N	1025	540	485
Age at diagnosis	Range 21–96 (median: 59)	Range 22–93 (median: 56)	Range 21–96 (median: 62)
Disease evolution			
Disease free	727	405	322
Metastasis	298	135	163
Stage at diagnosis (AJCC 8th edition)			
In situ	155	87	68
IA	367	204	163
IB	136	75	61
IIA	77	41	36
IIB	55	31	24
IIC	59	26	33
IIIA	31	13	18
IIIB	22	10	12
IIIC	57	20	37
IIID	3	0	3
IV	13	7	6
nd	50	26	24
Melanoma subtypes			
SSM	524	279	245
NM	153	76	77
LM	36	17	19
LMM	52	29	23
ALM	48	31	17
Others	17	9	8
nd	195	99	96
Breslow Thickness (mm)			
0	158	89	69
≤1	403	223	180
>1–2	176	91	85
>2–4	132	72	60
>4	116	46	70
nd	40	19	21
Location			
Head/Neck	158	70	88
Trunk	424	165	259
Upper limb	125	77	48
Lower limb	230	173	57
Hands/foot	56	39	17
Others	19	11	8

**Table 2:** Clinicopathological features of melanoma patients with at least 5 years of follow-up (n = 664). SSM, superficial spreading melanoma; NM, nodular melanoma; LM, lentigo maligna; LMM, lentigo maligna melanoma; ALM, acral lentiginous melanoma; nd: no data.

	All Patients	Women	Men
N	664	360	304
Age at diagnosis	Range 22–93 (median: 58)	Range 22–93 (median: 53)	Range 22–93 (median: 62)
Disease evolution			
Disease free	469	275	194
Metastasis	195	85	110
Stage at diagnosis (AJCC 8th edition)			
In situ	91	53	38
IA	238	135	103
IB	91	56	35
IIA	61	33	28
IIB	38	22	16
IIC	42	19	23
IIIA	17	7	10
IIIB	11	5	6
IIIC	46	16	30
IIID	3	0	3
IV	13	7	6
nd	13	7	6
Melanoma subtypes			
SSM	362	196	166
NM	100	53	47
LM	19	8	11
LMM	21	13	8
ALM	37	24	13
Others	29	17	13
nd	96	49	46
Breslow Thickness (mm)			
0	88	49	39
≤1	257	146	111
>1–2	116	64	52
>2–4	92	52	40
>4	85	33	52
nd	26	16	10
Location			
Head/Neck	86	35	51
Trunk	265	105	160
Upper limb	84	52	32
Lower limb	165	124	41
Hands/foot	42	31	11
Others	12	8	4
nd	10	5	5

**Table 3:** Genotype frequencies of rs1042602 in patients and controls. Chi-Square Test and Cochran–Armitage test for trend test (CATT).

	Genotype N (%)			Chi2 Test	CATT Test
	CC	CA	AA	*p*-Value	*p*-Value
All patients					
Controls	210 (27.2%)	373 (48.2%)	190 (24.6%)	0.0044	0.0035
Patients	221 (21.6%)	494 (48.2%)	310 (30.2%)		
Men					
Controls	65 (31%)	105 (50%)	40 (19%)	0.0015	0.0030
Patients	101 (20.8%)	238 (49.1%)	146 (30.1%)		
Women					
Controls	108 (24.7%)	207 (47.2%)	123 (28.1%)	0.5915	0.2061
Patients	120 (22.2%)	256 (47.4%)	164 (30.4%)		

**Table 4:** Statistical analyses of the differences in rs1042602 frequencies between metastatic and non-metastatic patients in all samples, and stratified by sex. Chi-Square Test and Cochran–Armitage test for trend test (CATT).

Samples	Chi-Square Test (df = 2) (*p*-Value)			CATT Test: One-Sided (Theta = 1) (*p*-Value)		
	ALL	WOMEN	MEN	ALL	WOMEN	MEN
All stages	0.1526	0.7118	0.0308	0.0384	0.5968	0.0054
Stages I, II and III	0.1188	0.4231	0.0287	0.0471	0.6761	0.0052
Stage II	0.4493	0.8737	0.1503	0.1541	0.6162	0.0716

**Table 5:** Genotype frequencies of rs1042602 in male patients with a follow-up period of 5 years, grouped by their prognosis (metastatic vs. disease-free). Chi-Square Test and Cochran–Armitage test for trend test (CATT).

	Genotype N (%)			Chi2 Test	CATT Test
	CC	CA	AA	*p*-Value	*p*-Value
All stages (n = 304)					
Disease-free	45 (23.2%)	91 (46.9%)	58 (29.9%)	0.0308	0.0054
Metastatic	12 (10.9%)	60 (54.55%)	38 (34.55%)		
Stages I, II and III (n = 254)					
Disease-free	34 (21.9%)	72 (46.5%)	49 (31.6%)	0.0287	0.0052
Metastatic	9 (9.1%)	53 (53.5%)	37 (37.4%)		
Stage II (n = 67)					
Disease-free	6 (23.1%)	12 (46.1%)	8 (30.8%)	0.1503	0.0716
Metastatic	3 (7.3%)	26 (63.4%)	12 (29.3%)		

**Table 6:** Cox’s proportional hazard model for disease-free survival (DFS) for rs1042602.

Samples		Univariate		Multivariate	
		HR (95% CI)	*p*-Value	HR (95% CI)	*p*-Value
Men and women (n = 1025)	CC	0.62 (0.42–0.93)	0.0202	0.64 (0.41–1.01)	0.0530
	CA	0.92 (0.69–1.22)	0.5579	0.86 (0.63–1.18)	0.3550
Men and women, stages I, II, III (n = 807)	CC	0.59 (0.38–0.89)	0.0145	0.62 (0.39–0.99)	0.0446
	CA	0.84 (0.63–1.14)	0.2616	0.85 (0.62–1.18)	0.3331
Men (n = 485)	CC	0.49 (0.27–0.89)	0.0183	0.48 (0.25–0.94)	0.0320
	CA	0.99 (0.68–1.44)	0.9545	0.85 (0.55–1.30)	0.4450
Men, stages I, II, III (n = 387)	CC	0.43 (0.23–0.82)	0.0099	0.44 (0.21–0.89)	0.0219
	CA	0.93 (0.63–1.38)	0.7278	0.86 (0.56–1.32)	0.4870
Men, 5 years follow-up (n = 304)	CC	0.46 (0.24–0.89)	0.0200	0.4 (0.20–0.83)	0.0139
	CA	0.95 (0.63–1.42)	0.7940	0.68 (0.42–1.11)	0.1218
Men, 5 years follow-up, stages I, II, III (n = 254)	CC	0.4 (0.19–0.83)	0.0136	0.36 (0.16–0.79)	0.0107
	CA	0.9 (0.59–1.37)	0.6191	0.7 (0.43–1.13)	0.1461

**Table 7:** Cox’s proportional hazard model for disease-free survival (DFS) for rs1042602, grouped as CC vs. CA and AA.

Samples	Univariate		Multivariate	
	HR (95% CI)	*p*-Value	HR (95% CI)	*p*-Value
Men and women (n = 1025)	0.66 (0.46–0.94)	0.0222	0.7 (0.47–1.06)	0.0904
Men and women, stages I, II, III (n = 807)	0.65 (0.44–0.96)	0.0308	0.68 (0.44–1.05)	0.0789
Men (n = 485)	0.50 (0.29–0.85)	0.0110	0.54 (0.29–0.99)	0.0459
Men, stages I, II, III (n = 387)	0.45 (0.25–0.82)	0.0086	0.48 (0.25–0.93)	0.0290
Men, 5 years follow-up (n = 304)	0.48 (0.26–0.87)	0.0160	0.51 (0.26–0.99)	0.0480
Men, 5 years follow-up, stages I, II, III (n = 254)	0.43 (0.21–0.85)	0.0147	0.45 (0.21–0.93)	0.0318

**Table 8:** Frequencies of rs1042602 genotypes according to melanoma staging (AJCC 8th edition) in patients with 5 years of follow-up.

	CC (%)	CA (%)	AA (%)	N
Men and women	19.4	49.2	31.4	651
In situ	24.2	46.1	29.7	91
Stage I	20.7	49.5	29.8	329
Stage II	16.3	51.1	32.6	141
Stage III	11.7	48.0	40.3	77
Stage IV	30.8	46.1	23.1	13
Men	18.8	49.0	32.2	298
In situ	26.3	50.0	23.7	38
Stage I	22.5	45.6	31.9	138
Stage II	13.4	56.7	29.9	67
Stage III	6.1	49.0	44.9	49
Stage IV	50.0	33.3	16.7	6
Women	19.8	49.3	30.9	353
In situ	22.6	43.4	34.0	53
Stage I	19.4	52.3	28.3	191
Stage II	18.9	46.0	35.1	74
Stage III	21.4	46.4	32.2	28
Stage IV	14.3	57.1	28.6	7

**Table 9:** Cox’s proportional hazard model for distant metastasis-free survival (DMFS) in patients with stage III melanomas and 5 years of follow-up.

	Univariate			Multivariate		
Variable	Coef	HR (95% CI)	*p*-Value	Coef	HR (95% CI)	*p*-Value
Men and women						
Age	0.0359	1.0365 (1.002–1.073)	0.0404	0.0520	1.0534 (1.015–1.093)	0.0061
Sex	0.9262	2.5249 (0.828–7.704)	0.1040	-	-	-
Breslow thickness	0.0980	1.1029 (0.992–1.226)	0.0693	-	-	-
Ulceration	0.8022	2.2305 (0.842–5.907)	0.1060	-	-	-
Melanoma subtypes	0.0077	1.0078 (0.680–1.494)	0.9690	-	-	-
rs1042602 genotype	−1.1387	0.3202 (0.127–0.809)	0.0160	−1.5605	0.2100 (0.079–0.555)	0.0017
Men						
Age	0.0340	1.0346 (0.997–1.074)	0.0719	-	-	-
Breslow thickness	0.0887	1.0928 (0.983–1.215)	0.1	-	-	-
Ulceration	1.1984	3.3148 (1.075–10.22)	0.037	1.7475	5.7401 (1.594–20.657)	0.0075
Melanoma subtypes	0.1234	1.1313 (0.752–1.702)	0.5540	-	-	-
rs1042602 genotype	−1.3253	0.2657 (0.090–0.787)	0.0168	−1.8709	0.154 (0.044- 0.539)	0.0034

Dash indicates not included in multivariate analysis due to lack of significance in univariate analysis. Coef, beta coefficient; HR, hazard ratio; CI, confidence interval.

## Data Availability

Not applicable.

## References

[1] Garbe C., Amaral T., Peris K., Hauschild A., Arenberger P., Basset-Seguin N., Bastholt L., Bataille V., Del Marmol V., Dréno B. (2022). European consensus-based interdisciplinary guideline for melanoma. Part 1: Diagnostics: Update 2022. Eur. J. Cancer.

[2] Whiteman D.C., Green A.C., Olsen C.M. (2016). The Growing Burden of Invasive Melanoma: Projections of Incidence Rates and Numbers of New Cases in Six Susceptible Populations through 2031. J. Investig. Dermatol..

[3] Gershenwald J.E., Scolyer R.A. (2018). Melanoma Staging: American Joint Committee on Cancer (AJCC) 8th Edition and Beyond. Ann. Surg. Oncol..

[4] Rendleman J., Shang S., Dominianni C., Shields J.F., Scanlon P., Adaniel C., Desrichard A., Ma M., Shapiro R., Berman R. (2013). Melanoma risk loci as determinants of melanoma recurrence and survival. J. Transl. Med..

[5] Vogelsang M., Wilson M., Kirchhoff T. (2016). Germline determinants of clinical outcome of cutaneous melanoma. Pigment Cell Melanoma Res..

[6] Rendleman J., Vogelsang M., Bapodra A., Adaniel C., Silva I., Moogk D., Martinez C.N., Fleming N., Shields J., Shapiro R. (2015). Genetic associations of the interleukin locus at 1q32.1 with clinical outcomes of cutaneous melanoma. J. Med. Genet..

[7] Vogelsang M., Martinez C.N., Rendleman J., Bapodra A., Malecek K., Romanchuk A., Kazlow E., Shapiro R.L., Berman R.S., Krogsgaard M. (2016). The Expression Quantitative Trait Loci in Immune Pathways and their Effect on Cutaneous Melanoma Prognosis. Clin. Cancer Res..

[8] Aoude L.G., Bonazzi V.F., Brosda S., Patel K., Koufariotis L.T., Oey H., Nones K., Wood S., Pearson J.V., Lonie J.M. (2020). Pathogenic germline variants are associated with poor survival in stage III/IV melanoma patients. Sci. Rep..

[9] López S., García O., Yurrebaso I., Flores C., Acosta-Herrera M., Chen H., Gardeazabal J., Careaga J.M., Boyano M.D., Sánchez A. (2014). The interplay between natural selection and susceptibility to melanoma on allele 374F of SLC45A2 gene in a South European population. PLoS ONE.

[10] García-Borrón J.C., Abdel-Malek Z., Jiménez-Cervantes C. (2014). MC1R, the CAMP Pathway, and the Response to Solar UV: Extending the Horizon beyond Pigmentation. Pigment Cell Melanoma Res..

[11] Ainger S.A., Jagirdar K., Lee K.J., Soyer H.P., Sturm R.A. (2017). Skin Pigmentation Genetics for the Clinic. Dermatology.

[12] Landi M.T., Bishop D.T., MacGregor S., Machiela M.J., Stratigos A.J., Ghiorzo P., Brossard M., Calista D., Choi J., Fargnoli M.C. (2020). Genome-wide association meta-analyses combining multiple risk phenotypes provide insights into the genetic architecture of cutaneous melanoma susceptibility. Nat. Genet..

[13] Lourenço G.J., Oliveira C., Carvalho B.S., Torricelli C., Silva J.K., Gomez G.V.B., Rinck-Junior J.A., Oliveira W.L., Vazquez V.L., Serrano S.V. (2020). Inherited variations in human pigmentation-related genes modulate cutaneous melanoma risk and clinicopathological features in Brazilian population. Sci. Rep..

[14] Weir B.S., Cockerham C.C. (1984). Estimating F-Statistics for the Analysis of Population Structure. Evolution.

[15] Danecek P., Auton A., Abecasis G., Albers C.A., Banks E., DePristo M.A., Handsaker R., Lunter G., Marth G., Sherry S.T. (2011). The Variant Call Format and VCFtools. Bioinformatics.

[16] Tian J., Xu C., Zhan H., Yang Y. (2009). Exact MAX Tests in Case-Control Association Analysis (Manuscript). https://rdrr.io/cran/MaXact/man/maxact.html.

[17] Zheng X., Levine D., Shen J., Gogarten S., Laurie C., Weir B. (2012). A High-performance Computing Toolset for Relatedness and Principal Component Analysis of SNP Data. Bioinformatics.

[18] Therneau T. (2022). A Package for Survival Analysis in R. R Package Version 3.3-1. https://CRAN.R-project.org/package=survival.

[19] Chen H., Conomos M.P., Pham D.T. (2021). GMMAT: Generalized LinearMixed Model Association Tests. R Package Version 1.3.2. https://rdrr.io/cran/GMMAT/.

[20] Shriver M.D., Parra E.J., Dios S., Bonilla C., Norton H., Jovel C., Pfaff C., Jones C., Massac A., Cameron N. (2003). Skin pigmentation, biogeographical ancestry and admixture mapping. Hum. Genet..

[21] Stokowski R.P., Pant P.V., Dadd T., Fereday A., Hinds D.A., Jarman C., Filsell W., Ginger R.S., Green M.R., van der Ouderaa F.J. (2007). A genomewide association study of skin pigmentation in a South Asian population. Am. J. Hum. Genet..

[22] Jonnalagadda M., Norton H., Ozarkar S., Kulkarni S., Ashma R. (2016). Association of genetic variants with skin pigmentation phenotype among populations of west Maharashtra, India. Am. J. Hum. Biol..

[23] Adhikari K., Mendoza-Revilla J., Sohail A., Fuentes-Guajardo M., Lampert J., Chacón-Duque J.C., Hurtado M., Villegas V., Granja V., Acuña-Alonzo V. (2019). A GWAS in Latin Americans highlights the convergent evolution of lighter skin pigmentation in Eurasia. Nat. Commun..

[24] Lona-Durazo F., Hernandez-Pacheco N., Fan S., Zhang T., Choi J., Kovacs M.A., Loftus S.K., Le P., Edwards M., Fortes-Lima C.A. (2019). Meta-analysis of GWA studies provides new insights on the genetic architecture of skin pigmentation in recently admixed populations. BMC Genet..

[25] Shan M.A., Meyer O.S., Refn M., Morling N., Andersen J.D., Børsting C. (2021). Analysis of Skin Pigmentation and Genetic Ancestry in Three Subpopulations from Pakistan: Punjabi, Pashtun, and Baloch. Genes.

[26] Frudakis T., Thomas M., Gaskin Z., Venkateswarlu K., Chandra K.S., Ginjupalli S., Gunturi S., Natrajan S., Ponnuswamy V.K., Ponnuswamy K.N. (2003). Sequences associated with human iris pigmentation. Genetics.

[27] Morgan M.D., Pairo-Castineira E., Rawlik K., Canela-Xandri O., Rees J., Sims D., Tenesa A., Jackson I.J. (2018). Genome-wide study of hair colour in UK Biobank explains most of the SNP heritability. Nat. Commun..

[28] Sulem P., Gudbjartsson D.F., Stacey S.N., Helgason A., Rafnar T., Magnusson K.P., Manolescu A., Karason A., Palsson A., Thorleifsson G. (2007). Genetic determinants of hair.; eye and skin pigmentation in Europeans. Nat. Genet..

[29] Galván-Femenía I., Obón-Santacana M., Piñeyro D., Guindo-Martinez M., Duran X., Carreras A., Pluvinet R., Velasco J., Ramos L., Aussó S. (2018). Multitrait genome association analysis identifies new susceptibility genes for human anthropometric variation in the GCAT cohort. J. Med. Genet..

[30] Thomas N.E., Kricker A., Waxweiler W.T., Dillon P.M., Busman K.J., From L., Groben P.A., Armstrong B.K., Anton-Culver H., Gruber S.B. (2014). Comparison of clinicopathologic features and survival of histopathologically amelanotic and pigmented melanomas: A population-based study. JAMA Dermatol..

[31] Guo W., Yin G., Liu H., Duan H., Huang Z., Chen X. (2020). Matched analysis of the prognosis of amelanotic and pigmented melanoma in head and neck. Acta Otolaryngol..

[32] Ryu G.W., Choi Y.D., Jin S., Chung I.J., Shin M.H., Yun S.J. (2021). Volar location and degree of pigmentation are associated with poor survival and first metastasis pattern in acral melanoma. Pigment Cell Melanoma Res..

[33] Giebel L.B., Spritz R.A. (1990). RFLP for MboI in the human tyrosinase (TYR) gene detected by PCR. Nucleic Acids Res..

[34] Ito S. (2003). The IFPCS presidential lecture: A chemist’s view of melanogenesis. Pigment Cell Res..

[35] Chaki M., Sengupta M., Mondal M., Bhattacharya A., Mallick S., Bhadra R., Ray K., Indian Genome Variation Consortium (2011). Molecular and functional studies of tyrosinase variants among Indian oculocutaneous albinism type 1 patients. J. Investig. Dermatol..

[36] Jagirdar K., Smit D.J., Ainger S.A., Lee K.J., Brown D.L., Chapman B., Zhao Z.Z., Montgomery G.W., Martin N.G., Stow J.L. (2014). Molecular analysis of common polymorphisms within the human Tyrosinase locus and genetic association with pigmentation traits. Pigment Cell Melanoma Res..

[37] Hoggart C.J., Parra E.J., Shriver M.D., Bonilla C., Kittles R.A., Clayton D.G., McKeigue P.M. (2003). Control of confounding of genetic associations in stratified populations. Am. J. Hum. Genet..

[38] Chaitanya L., Breslin K., Zuñiga S., Wirken L., Pośpiech E., Kukla-Bartoszek M., Sijen T., Knijff P., Liu F., Branicki W. (2018). The HIrisPlex-S system for eye, hair and skin colour prediction from DNA: Introduction and forensic developmental validation. Forensic Sci. Int. Genet..

[39] Kalahroudi V.G., Kamalidehghan B., Kani A.A., Aryani O., Tondar M., Ahmadipour F., Chung L.Y., Houshmand M. (2014). Two novel tyrosinase (TYR) gene mutations with pathogenic impact on oculocutaneous albinism type 1 (OCA1). PLoS ONE.

[40] Grønskov K., Jespersgaard C., Bruun G.H., Harris P., Brøndum-Nielsen K., Andresen B.S., Rosenberg T. (2019). A pathogenic haplotype, common in Europeans, causes autosomal recessive albinism and uncovers missing heritability in OCA1. Sci. Rep..

[41] Mendez R., Iqbal S., Vishnopolska S., Martinez C., Dibner G., Aliano R., Zaiat J., Biagioli G., Fernandez C., Turjanski A. (2021). Oculocutaneous albinism type 1B associated with a functionally significant tyrosinase gene polymorphism detected with Whole Exome Sequencing. Ophthalmic Genet..

[42] Bishop D.T., Demenais F., Iles M.M., Harland M., Taylor J.C., Corda E., Randerson-Moor J., Aitken J.F., Avril M.F., Azizi E. (2009). Genome-wide association study identifies three loci associated with melanoma risk. Nat. Genet..

[43] Amos C.I., Wang L.E., Lee J.E., Gershenwald J.E., Chen W.V., Fang S., Kosoy R., Zhang M., Qureshi A.A., Vattathil S. (2011). Genome-wide association study identifies novel loci predisposing to cutaneous melanoma. Hum. Mol. Genet..

[44] Hernando B., Ibarrola-Villava M., Fernandez L.P., Peña-Chilet M., Llorca-Cardeñosa M., Oltra S.S., Alonso S., Boyano M.D., Martinez-Cadenas C., Ribas G. (2016). Sex-specific genetic effects associated with pigmentation, sensitivity to sunlight, and melanoma in a population of Spanish origin. Biol. Sex Differ..

[45] Duffy D.L., Zhao Z.Z., Sturm R.A., Hayward N.K., Martin N.G., Montgomery G.W. (2010). Multiple pigmentation gene polymorphisms account for a substantial proportion of risk of cutaneous malignant melanoma. J. Investig. Dermatol..

[46] Castaneda-Garcia C., Iyer V., Nsengimana J., Trower A., Droop A., Brown K.M., Choi J., Zhang T., Harland M., Newton-Bishop J.A. (2022). Defining novel causal SNPs and linked phenotypes at melanoma-associated loci. Hum. Mol. Genet..

[47] Takeuchi H., Kuo C., Morton D.L., Wang H.J., Hoon D.S. (2003). Expression of differentiation melanoma-associated antigen genes is associated with favorable disease outcome in advanced-stage melanomas. Cancer Res..

[48] Parlar A., Sayitoglu E.C., Ozkazanc D., Georgoudaki A.M., Pamukcu C., Aras M., Josey B.J., Chrobok M., Branecki S., Zahedimaram P. (2019). Engineering antigen-specific NK cell lines against the melanoma-associated antigen tyrosinase via TCR gene transfer. Eur. J. Immunol..

[49] Fürst K., Steder M., Logotheti S., Angerilli A., Spitschak A., Marquardt S., Schumacher T., Engelmann D., Herchenröder O., Rupp R.A.W. (2019). DNp73-induced degradation of tyrosinase links depigmentation with EMT-driven melanoma progression. Cancer Lett..

[50] Kamo H., Kawahara R., Simizu S. (2022). Tyrosinase suppresses vasculogenic mimicry in human melanoma cells. Oncol. Lett..

[51] Millán-Esteban D., Peña-Chilet M., García-Casado Z., Manrique-Silva E., Requena C., Bañuls J., López-Guerrero J.A., Rodríguez-Hernández A., Traves V., Dopazo J. (2021). Mutational Characterization of Cutaneous Melanoma Supports Divergent Pathways Model for Melanoma Development. Cancers.

